# Inferring interactions in multispecies communities: The cryptocurrency market case

**DOI:** 10.1371/journal.pone.0291130

**Published:** 2023-09-15

**Authors:** E. Brigatti, V. Rocha Grecco, A. R. Hernández, M. A. Bertella

**Affiliations:** 1 Instituto de Física, Universidade Federal do Rio de Janeiro, Rio de Janeiro, RJ, Brazil; 2 Department of Economics, São Paulo State University (UNESP), Araraquara, SP, Brazil; URV: Universitat Rovira i Virgili, SPAIN

## Abstract

We introduce a general framework for empirically detecting interactions in communities of entities characterized by different features. This approach is inspired by ideas and methods coming from ecology and finance and is applied to a large dataset extracted from the cryptocurrency market. The inter-species interaction network is constructed using a similarity measure based on the log-growth rate of the capitalizations of the cryptocurrency market. The detected relevant interactions are only of the cooperative type, and the network presents a well-defined clustered structure, with two practically disjointed communities. The first one is made up of highly capitalized cryptocurrencies that are tightly connected, and the second one is made up of small-cap cryptocurrencies that are loosely linked. This approach based on the log-growth rate, instead of the conventional price returns, seems to enhance the discriminative potential of the network representation, highlighting a modular structure with compact communities and a rich hierarchy that can be ascribed to different functional groups. In fact, inside the community of the more capitalized coins, we can distinguish between clusters composed of some of the more popular first-generation cryptocurrencies, and clusters made up of second-generation cryptocurrencies. Alternatively, we construct the network of directed interactions by using the partial correlations of the log-growth rate. This network displays the important centrality of Bitcoin, discloses a core cluster containing a branch with the most capitalized first-generation cryptocurrencies, and emphasizes interesting correspondences between the detected direct pair interactions and specific features of the related currencies. As risk strongly depends on the interaction structure of the cryptocurrency system, these results can be useful for assisting in hedging risks. The inferred network topology suggests fewer probable widespread contagions. Moreover, as the riskier coins do not strongly interact with the others, it is more difficult that they can drive the market to more fragile states.

## Introduction

Identifying interactions in a community where a large number of entities of different types interact is an important and not obvious task in various areas of knowledge. Historically, this is a central problem for ecology and conservation biology [1]. Studies in these areas have tried to introduce robust and general methods for estimating pairwise species interactions [2]. There is a reach literature where estimation of species interactions is realized through field or laboratory observations where consumer-resource trophic interactions in empirical food webs are highlighted, or in experiments that compare variation in species abundance after deleting species. These approaches are difficult to be generalized to non biological systems. For these situations, the methods which use theoretical models, usually based on generalized Lotka-Volterra equations, can be more interesting. Here, the interaction strengths may be captured by characterizing the responses to a small perturbation near an equilibrium point [3]. Otherwise, interspecific interactions can be obtained by fitting the abundance or biomass time series of the species through a Lotka-Volterra, or analogous, model [4]. From these approaches it may be possible to identify predator-prey, competitive or mutualistic interactions but also amensalism/commensalism or situations of non-interacting species, where the population dynamics is neutral.

An alternative method for inferring the strength of interactions makes use of the Maximum Entropy Framework (MEF) [5]. In this approach, once obtained the covariance matrix from the abundance data, the elements of the negative inverse of this matrix can be naturally interpreted as the interactions between species. This interpretation has been confirmed by using a stochastic model analog to Lotka-Volterra, from which interactions have been inferred through a fitting procedure on the abundances [5]. By definition, the elements of this effective interaction matrix are the direct pairwise species interactions that reproduce the species covariances while maximizing the entropy of the system. The magnitude of these elements is a measure of the strength of the net interaction between a pair of species. The sign indicates the nature of the coupling: when positive, a change in the abundance of a given species generates a similar change in the abundance of the coupled one, when negative, a change in the first is followed by an opposite change in the second species. Since the considered matrix is symmetric by construction, only competitive (negative sign) or mutualistic (positive sign) interactions can be described.

It is worth noting that a traditional approach for characterizing dependence structures in ecological communities is based on the inspection of the covariances of species abundances [6, 7]. The MEF goes beyond the simple use of the covariances. In fact, in systems with many species there is no simple relationship between the covariance of a pair of species and their direct interaction. In fact, correlations can arise due to indirect interactions between two species through intermediate species or from common external drivers (e.g., two species can have a tenuous interaction and yet have a large correlation because they are both strongly interacting with a third one).

It is surprising that, operationally, the MEF for estimating pairwise species interactions is equivalent, up to a normalization, to a well-known method for numerically estimating partial correlations [8]. For definition, partial correlations measure the degree of association between two entities, removing the indirect effects of all the others. For this reason, they are an estimation of direct interactions, and they can approximate causal effects between components of a community better than correlations.

The characterization of couplings between components of markets is also a very important issue in finance [9]. The classical approach measures the correlation matrix between pairs of assets characterized by their return time series. The study of these matrices has a long history in finance. The spectral properties of these empirical correlation matrices are compared with random correlation matrices, to evidence the deviations from the null hypothesis (random matrix) [10], which can be considered as true information. A very active research direction represents these results by means of correlation networks where their topological, connectivity, and clustering properties are analyzed [11, 12]. In finance, the focus is more on the importance that correlations have in empirical applications than on the characterization of interactions and structures of the market. These matrices are related with fundamental applications in the theory of optimal portfolio [13]. Strong cross-correlations between the fluctuations of different assets modify the composition of an optimal portfolio and make diversification difficult.

The interest in describing dependencies among elements of a market has recently grown and encompassed the specific case of the cryptocurrency market [11], where new and original approaches have been introduced. For example, we can cite the work of Mensi *et al*. [14], which investigated multiscale relationships and causality between six prominent cryptocurrencies at 15-min intervals. Rolling window wavelet correlation analysis shows favorable co-movements and long-term memory across cryptocurrencies, particularly Bitcoin, Ethereum, and Monero. Most cryptocurrency pairs have dual causality, according to non-linear Granger causality tests. Hasan *et al*. [15] studied cryptocurrency market connectedness considering skewness and kurtosis. By using 5-minute data for eight of the top cryptocurrencies, the authors observe a robust asymmetry connection between Bitcoin, Ethereum and Litecoin, while a strong kurtosis relation is found between Bitcoin and Ethereum. Interestingly, Scagliarini *et al*. [16] analyzed higher-order dependencies in the cryptocurrency trading network. They examined the price returns of the cryptocurrency network utilizing both pairwise and high order statistical dependencies, as evaluated by Granger causality tests and O-information. The obtained network structure is relatively stable, and the coins are the most influential nodes. In the pairwise description of the network, stable coins seem to play a marginal role while they play a major role if high-order dependencies are considered.

In the present study, we will take inspiration from ideas and methods coming from the two different traditions of ecology and finance [17] to introduce a general framework for empirically detecting interactions in communities of entities characterized by different features (species). We will test this approach using an empirical dataset coming from the cryptocurrency market. This dataset generated by a synthetic community, in contrast with ecological data which are affected by sampling problems and limited statistics, has the advantage of presenting complete, accessible and rich statistics. It is a good example of a lively, spontaneous, big community with fast dynamics of creation and extinction of new classes of entities and with a temporal evolution characterized by different regimes [18]. Moreover, a natural parallelism between ecological systems and the cryptocurrency market can be constructed by considering that each cryptocurrency represents a species, and its capitalization its abundance. In this way, the wealth invested in a cryptocurrency replaces the population size of a species [18].

Once this analogy is introduced, it is natural to employ some ideas from ecological studies, such as the application of the MEF to data of abundance. As stated before, operationally, this method is equivalent to measuring the partial correlations of the considered stochastic variables. Using abundance time series (capitalization) can be difficult because this observable is frequently non-stationary and subject to large fluctuations and measurement errors. As it is usually done in finance, it is more robust to consider the fluctuation of the observable, which, in this case, corresponds to the difference between two consecutive logarithms of the abundance. This new observable is, in general, close to stationarity. Indeed, if the process has multiplicative dynamics, logarithms are a natural choice because they eliminate underlying exponential growth trends. Differencing is another simple method which help in reducing time-dependence in statistics. Moreover, this quantity has a clear and relevant empirical interpretation, corresponding to the definition of the logarithmic growth rate [19]. Since $\text{log}\left(c\left(t+1\right)\right)-\text{log}\left(c\left(t\right)\right)\approx \frac{1}{c}\frac{dc}{dt}$, this is also a common discrete approximation for the instantaneous per capita growth rate.

Finally, as commonly done in finance, we will represent the ensemble of detected interactions using interaction networks, which capture the particular structure of the interconnections and highlight the modular structure of the considered communities. A detailed description of the whole approach will be given in the next section.

## Methods

Networks are a very powerful method for representing dataset structures. Once the mapping between the dataset and networks is realized, a rich ensemble of information can be extracted. Dataset can be translated into a network by defining the network nodes and applying some criteria for introducing links between these nodes. These criteria are based on quantifying similarity between nodes, which can be obtained using cosine similarity, correlation, mutual information, or other dependence measures applied to the vectors, or time series, associated with two given nodes. Networks are finally constructed by linking two nodes whenever the similarity index is greater than a given threshold. Weighted networks can be generated by taking these indices as weights.

A more rigorous approach consists in constructing a distance, which satisfies the triangular inequality, from the similarity measure. This is generally obtained by introducing a function of the considered dependence measure. Once obtained the distance matrix, which collects the distances between all the considered nodes, one can build the final network representation by connecting nodes presenting shorter distances. The most straightforward method links two nodes whenever the distance is smaller than a given threshold. Otherwise, it is possible to build a tree representation using phylogenetic trees [20, 21] or spanning trees [22]. In general, we will follow this last method in our work.

The nodes of interaction networks represent species and, in our specific case, different cryptocurrencies. Links are generated from a correlation measure between the time series of a specific quantity which characterizes each node. The quantity commonly employed in finance is the return of a given cryptocurrency, defined as *R*(*t*) = log(*p*(*t* + 1)) − log(*p*(*t*)), where *p* is the price of the cryptocurrency at the time *t*. In our analysis, as stated before, we focus on the capitalization, and not the price, defining the logarithmic growth rate of the capitalization as:

$$\begin{array}{c} G(t)=\text{log}\;\left(c(t+1)\right)-\text{log}\;\left(c(t)\right), \end{array}$$ (1)

where *c* is the capitalization of the cryptocurrency at time *t*.

To avoid biases data are normalized by standardization:

$$\begin{array}{c} G_{n}\left(t\right)=\frac{G(t)-\langle G\rangle }{\sigma_{G}} \end{array}$$ (2)

where 〈〉 is the mean value and *σ* the standard deviation estimated over the whole time series. This transformation generates time series with zero means and unit standard deviation.

The similarity is estimated by applying Pearson’s correlation to the time series of the normalized log-growth rates: *C*_*ij*_ = *corr*(*G*_*i*_(*t*), *G*_*j*_(*t*)) for all the considered pairs of cryptocurrency *i* and *j*. *C*_*ij*_ happens to be the scalar product of the normalized time series *G*_*n*_. Consequently, we can define a distance *d*_*ij*_ in terms of this scalar product: $d_{ij}^{2}=|G_{i}-G_{j}{|}^{2}=|G_{i}{|}^{2}+{\left|G_{j}\right|}^{2}-2G_{i}\cdot G_{j}.$ By considering the normalization of *G*_*n*_, it reduces to [22]:

$$\begin{array}{c} d_{ij}=\sqrt{2(1-C_{ij})}. \end{array}$$ (3)

From its construction, this definition satisfies the triangular inequality, is symmetric, positive (0 ≤ *d*_*ij*_ ≤ 2) and zero only if *i* = *j*.

Once calculated the distance matrix, a tree representation is obtained by generating a Minimum Spanning Tree (MiST) [22] connecting the considered cryptocurrencies. As a MiST is the tree connecting all nodes with a minimum total weight, it is the backbone connecting the cryptocurrencies interacting with higher positive correlation values. If we want to highlight the cryptocurrencies characterized by large negative correlations, a Maximum Spanning Tree must be generated.

Considering that interactions are not necessarily instantaneous but, instead, can be mediated by a time delay, we also explored if time-lagged correlations can display higher values than unshifted ones.

Correlations allow to obtain a dependence measure between pairs of cryptocurrencies, but the network links identified using correlations can be due to indirect interactions between two cryptocurrencies through intermediate currencies. For example, two cryptocurrencies that have not interact directly still present a correlation because they interact with a third common coin. The removal of these indirect effects can be obtained measuring the pairwise partial correlations. In fact, the partial correlation between the time series of cryptocurrencies *i* and *j* is obtained removing the indirect effects of all the other nodes. This can be achieved by estimating the residuals of the linear least-squares predictor of their time series based on all the other cryptocurrencies, excluding the pair *i* and *j* [8]. An alternative approach calculates pairwise partial correlations from the inverse of the covariance matrix *Cov*, the so called precision matrix *P* = *Cov*^−1^. In detail, partial correlations *ρ*_*ij*_ are obtained from the negative of the precision matrix elements *P*_*ij*_ after normalization [8]:

$$\begin{array}{c} \rho_{ij}=-\frac{P_{ij}}{\sqrt{P_{ii}P_{jj}}}. \end{array}$$ (4)

Note that the diagonal of this matrix presents negative entries. It can be shown that the elements of −*ρ*_*ij*_ are related to a scalar product (of the elements in the dual basis of the vectors *G*_*n*_) which, this time, renders the following distance expression:

$$\begin{array}{c} d_{ij}=\sqrt{2(1+\rho_{ij}).} \end{array}$$ (5)

For this metric, a spanning tree which connects the cryptocurrencies with higher positive partial correlation values is displayed by a Maximum Spanning Tree (MaST), and not a MiST.

## Data

We analyze cryptocurrency data obtained from the website Coin Market Cap [23], which makes available from the exchange market platforms the price expressed in US dollars (exchange rate) and the market capitalization of the different cryptocurrencies. A capitalization of a single digital currency is calculated by multiplying its total number in circulation by its current market price. The importance of this concept can be understood by making an analogy with the stock market. The seminal work by Sharpe [24] and Lintner [25], who developed the Capital Asset Pricing Model, explains that stock returns are a function of market risk. Other researchers carried out empirical research to identify characteristics that could better explain the behavior of stock returns. One of these explanatory variables was firm size (or firm capitalization), found by Banz [26]. The firm size effect (or capitalization effect) refers to the finding that smaller firms have greater returns than larger companies. According to the hypothesis underlying this phenomena, a company’s size serves as a proxy for risk, with smaller firms being more risky than bigger ones, putting downward pressure on their share prices, generating higher returns for the investors.

We focus our analysis on daily data for the 100 cryptocurrencies with the largest capitalization. In this dataset, the capitalization time series of eight cryptocurrencies displays some lacunae. The coin LEO, which presents long intervals with no data, has been excluded from the analysis. The remaining coins have been included in the dataset after filling the lacunae with the average between the values available at the extremes of each gap.

The dataset is composed of daily data from 1 October 2015 to 31 March 2019, which corresponds to a series of 1278 time steps. The selection of this period is particularly suitable for our study. In this analysis, we are interested in characterizing the dynamics of interaction between the first and second generations of cryptocurrencies. The first generation of cryptocurrencies includes Bitcoin (BTC) [27] and other older coins that are generally Bitcoin forks—like Litecoin (LTC) and Dogecoin (DOGE). Most of them use a proof of work consensus mechanism and have a fully functional chain older than 2014. Around 2014, new projects emerge, distinguished by concepts that set them apart from every other cryptocurrency related to Bitcoin. These second generation cryptocurrencies present far more complex algorithms which allow to run smart contract functionalities. Ethereum (ETH) is the most successful coin of this generation. In recent years, new ideas and features have introduced the discussion on the possible presence of third and fourth generation cryptocurrencies. In this study we are not interested in analyzing this last, not well established, stage of cryptocurrency evolution. For this reason, we restrict our study to the data collected until 2019. Finally, we do not consider the first years of activity of the cryptocurrency market, characterized by a mass radiation phase, with a spectacular increase in the number of cryptocurrencies, and extreme fluctuations [18, 27].

## Results

A descriptive statistic of the log-growth rate time series shows a behavior analogous to the classical returns time series. We can reasonably approximate the time series as stationary and observe that data distributions present positive excess kurtosis (leptokurtic distributions).

**Fig 1:**
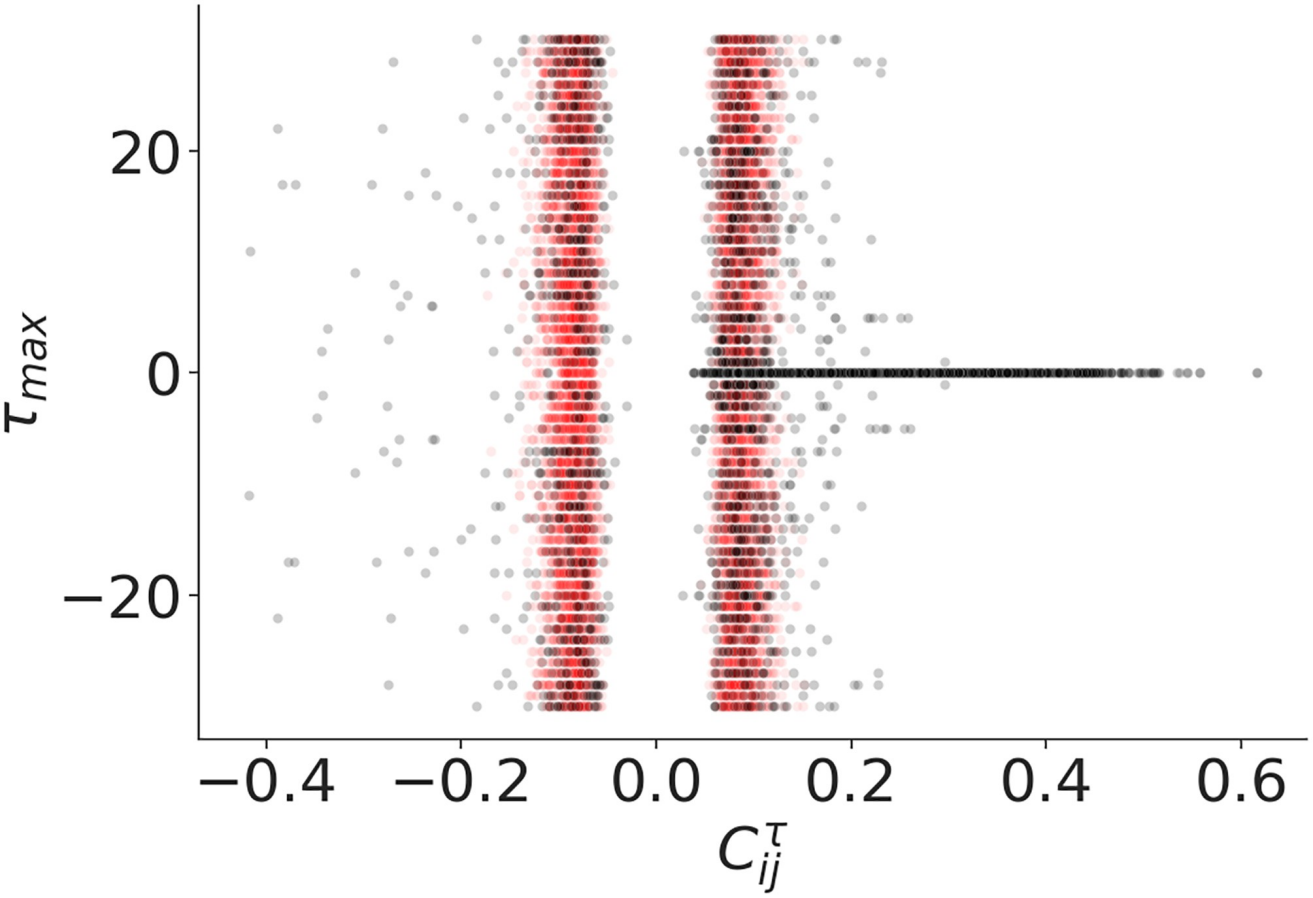
Scattering plot of *corr*(*G*_*i*_(*t* + *τ*_*max*_), *G*_*j*_(*t*)) versus *τ*_*max*_ for each pair of the considered cryptocurrencies. The black points are the original data and the red ones correspond to randomized data; *τ*_*max*_ = 0 in 82% of the original measurements.

First, we analyzed our dataset to discover if the strongest correlations between cryptocurrencies could appear at a later date. For each pair of coins, we calculate the cross-correlation $C_{ij}^{\tau }=corr\left(G_{i}\left(t+\tau \right),G_{j}\left(t\right)\right)$ between the two time series for time lags *τ* between 0 and 30 weeks. We observed the *τ* values where the absolute value of $C_{ij}^{\tau }$ reaches its maximum (*τ*_*max*_). Fig 1 shows the scattering plot of the largest $C_{ij}^{\tau_{max}}$ (maximum or minimum) of correlation versus the corresponding *τ*_*max*_. The comparison between real and randomized data indicates that, in general, the most relevant correlations are positive and generated at *τ* = 0. Due to this, in the analysis that follows, we measure correlation without considering temporal delay and we concentrate on positive correlations for the construction of networks.

**Fig 2:**
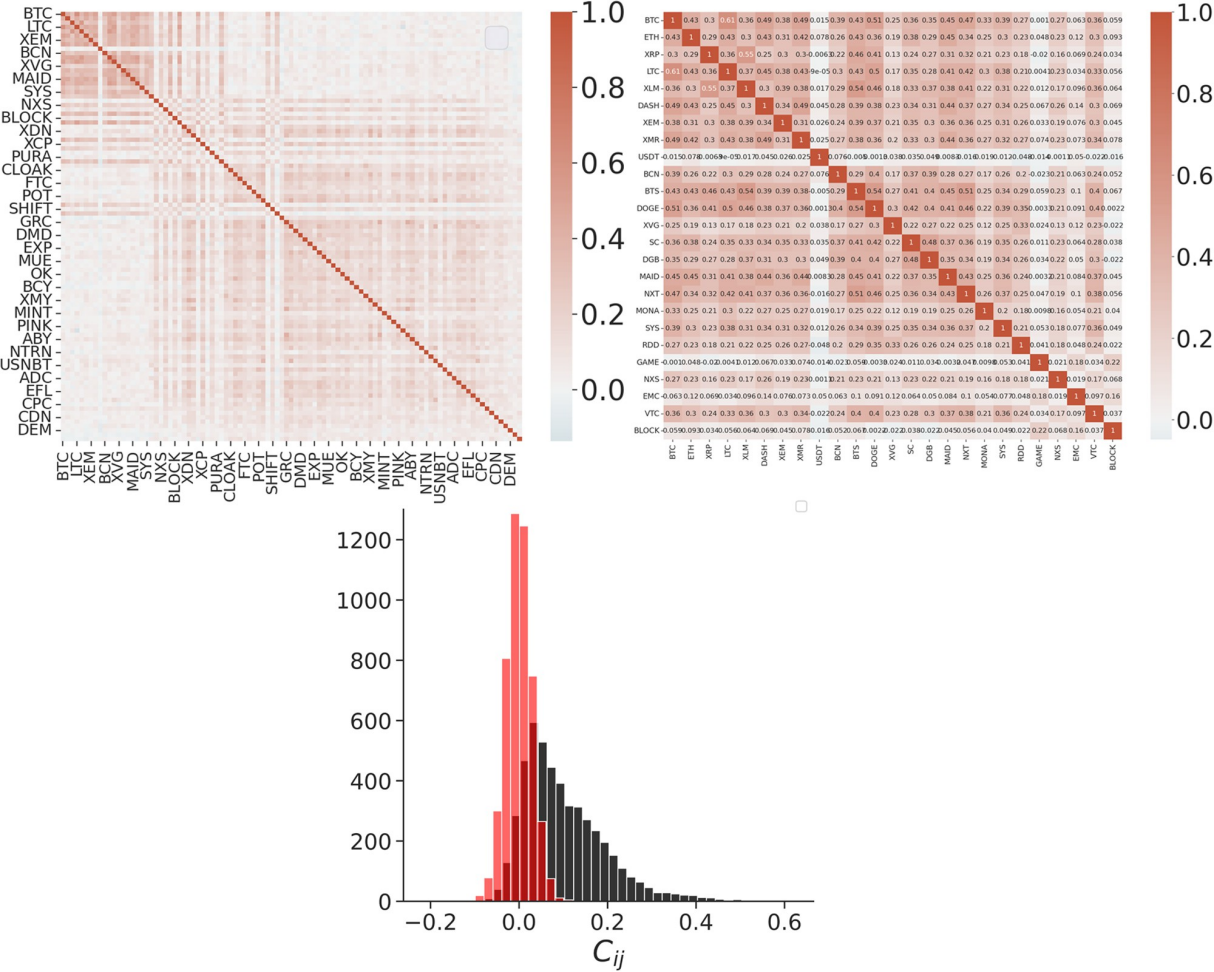
Correlation matrix of the log-growth rates for all the considered cryptocurrencies (left) and for the top 25 (right). This subset includes the block of the more capitalized highly-correlated cryptocurrencies. As the cryptocurrencies fill the matrix from the largest to the smallest capitalized ones, the 25 × 25 subset includes the block of the more capitalized highly-correlated cryptocurrencies. Note the block structure of the matrix, with the pairs of highly capitalized cryptocurrencies presenting higher correlations. Bottom: the distribution of the pair correlations for all the considered cryptocurrencies. In red, is the same distribution obtained from the randomized series.

We displayed the correlation matrix for the pairs of considered cryptocurrencies (see Fig 2), ordering the currencies based on their total capitalization. Surprisingly, the matrix shows a clear block structure, close to a block diagonal matrix. A first and smaller block contains the correlations produced by pairs of cryptocurrencies with higher capitalization, which present the larger values. The only exception among these highly capitalized coins is the USD Tether, a stablecoin pegged to the US dollar, which presents small correlations with the other cryptocurrencies. It is obvious that we are very far from a random matrix. Comparing the distribution of the pair correlations with the same distribution obtained from random shuffled series shows that a relevant number of statistically significant positive high-correlated pairs exists.

**Fig 3:**
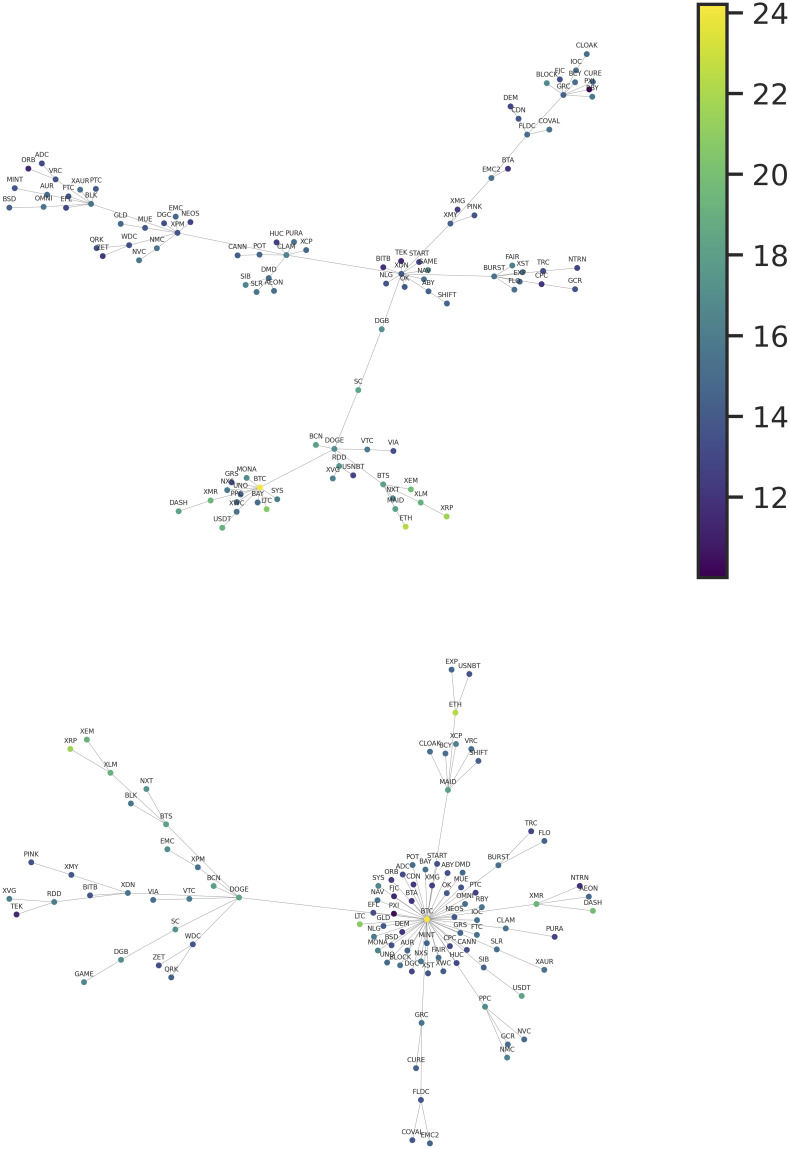
Top: Minimal spanning trees for the cryptocurrency market constructed using the pair correlations of the time series of daily log-growth rates. Bottom: Minimal spanning trees for the cryptocurrency market constructed using the pair correlations of the time series of daily returns. In the two networks, each node color represents the total capitalization of the corresponding cryptocurrency.

These features of the matrix suggest that a network representation of this dataset should be characterized by something close to the union of two disjoint graphs. We can see this structure by looking at Fig 3, which represents the MiST generated using the distance in 3. As practically only the positive correlations are statistically significant, this is the correct representation to highlight positive interactions among the community of cryptocurrencies. This tree presents a clear community structure, with two practically disjoint large communities. The first, and smaller one, is characterized by the currencies of higher capitalization, and the second, larger one, by the currencies of lower capitalization.

Descending the hierarchy of clusters, in the community of cryptocurrencies with higher capitalization, we can identify two main clusters. One cluster grew up around the BTC. This cluster is mainly composed of coins intended to be peer-to-peer electronic cash (first generation cryptocurrencies). Here, besides BTC, the most notable coins are Litecoin (LTC), DASH, and Monero (XMR). The first one is a fork (clone) of BTC, adjusted in order to be lighter and provide a cheaper transaction cost. DASH and XMR present privacy-enhancing technologies that obfuscate transactions in order to achieve anonymity and fungibility. It is also noticeable an interesting cluster, composed by second-generation cryptocurrencies, starting at the node of the BitShares (BTS) currency, which is arguably the first blockchain to allow for the implementation of decentralized finance. In this cluster, two other important coins, ETH and XRP, can be found at the end of two different branches. The XRP branch is composed of coins that continue the proposal of BTS by providing decentralized exchange capabilities. In contrast, the ETH branch is related to more general programmability, i.e., decentralized virtual machines capable of deploying and processing smart contracts. Curiously, these clusters are connected to DOGE, a highly-capitalized and very popular meme-coin that has neither technological proposal nor a development team. DOGE central position may be due to the currency popularity as a speculative asset.

For comparison, Fig 3 also displays the MiST produced using the same framework but with the returns of the prices of the cryptocurrencies instead of the log-growth rates. This is a classical approach used for characterizing a financial market and the results have already been presented in previous works [11]. Even if the capitalization and the price of the cryptocurrencies are obviously strongly correlated quantities, it is impressive how the topological structure of the two MiST is deeply different. In the MiST generated from the price returns, we can observe the strong centrality of the BTC, which interacts with a cloud of coins with small capitalization. The other high-capitalized coins are scattered in the tree, with two minor, more disjointed clusters, containing ETH and DOGE.

**Fig 4:**
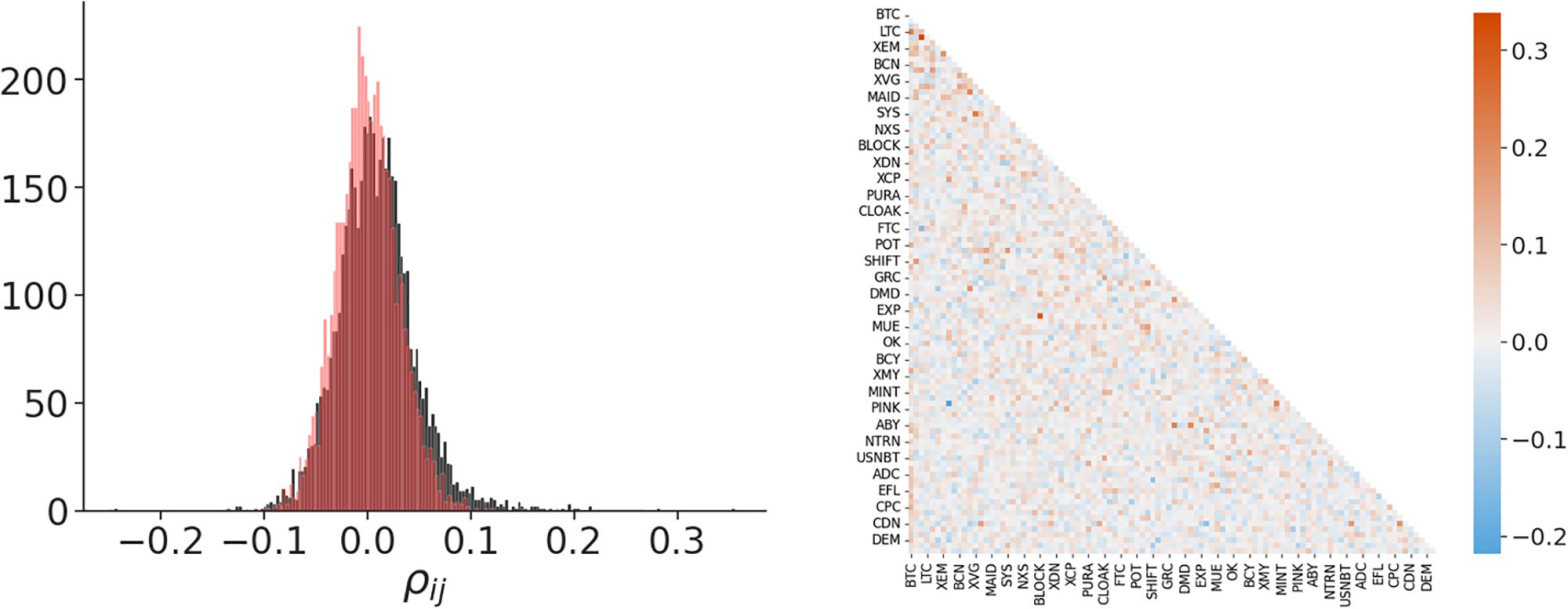
The distributions of the partial correlations *ρ*_*ij*_ for pairs of cryptocurrencies with *i* > *j*. In red are the same distributions obtained from the randomly shuffled series. The partial auto-correlations, obviously, have values equal to -1. Interspecific partial correlations can be both positive or negative, with values ranging from -0.24 and 0.35. On the left: the partial-correlation matrix of the growth rates for the pairs with *i* > *j*. Cryptocurrencies fill the matrix from the largest to the smallest capitalized ones.

Fig 4 shows the distribution of the partial correlations for all the considered pairs of cryptocurrencies. Comparing this distribution with the one obtained from the random shuffled series, it is evident that a relevant number of pairs with statistically significant, high-positive partial correlations can be outlined. The partial correlation matrix does not present a clear structure, as in the case of the correlations, but a more pronounced concentration of cryptocurrencies with higher partial correlation can be evidenced among the ones with higher capitalization (see Fig 4).

**Fig 5:**
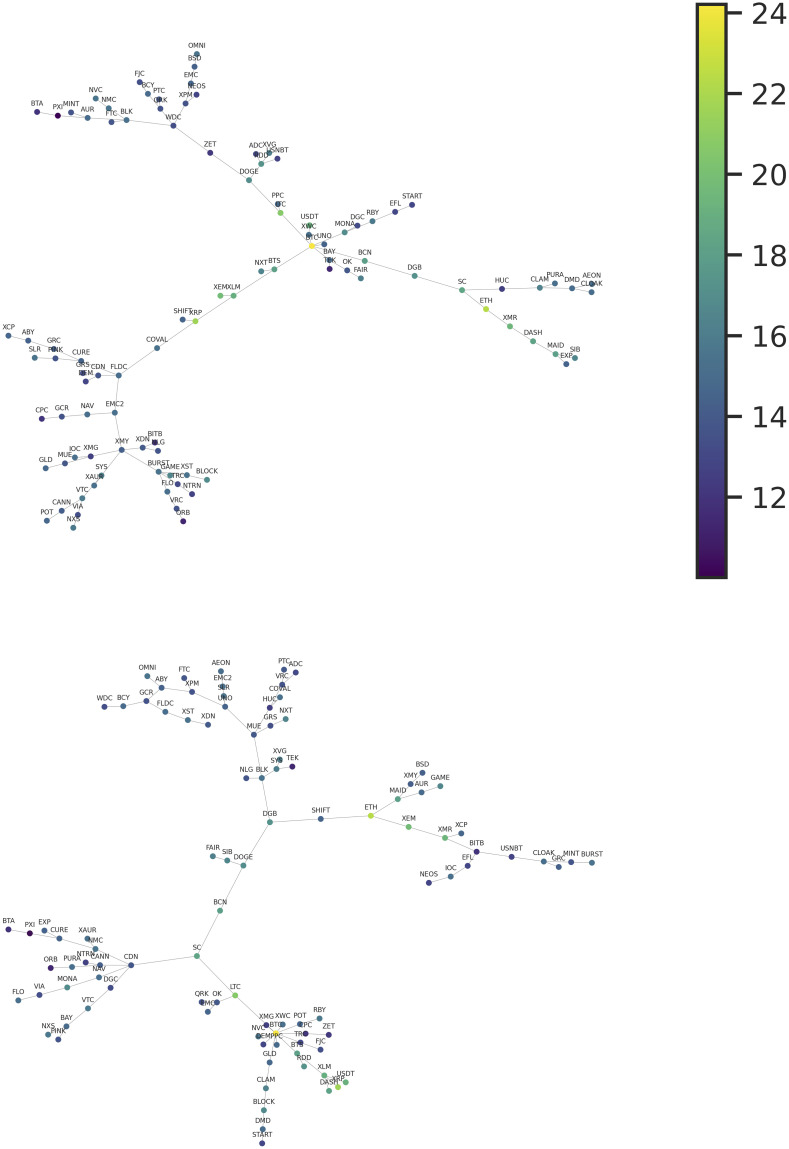
Top: Maximum spanning tree built using the partial correlations of the time series of daily log-growth rates. Bottom: MaST constructed using the partial correlations of the time series of daily price returns. In the two networks, each node color represents the total capitalization of the corresponding cryptocurrency.

Also in this case, we will focus our analysis only on the positive partial correlations, as in this case the pairs which display statistically significant values are numerically consistent. The Maximal Spanning Tree generated from the distance constructed by using the partial correlations of the log-growth rate (see Eq 5) is displayed in Fig 5. Here, the tree loses the highly ramified clusters and it becomes closer to a hierarchical sum of linear chains. This fact is expected as the links representing direct interactions are reduced in relation to links constructed using Pearson’s correlation. It is worth noting that, also in this case, coins with different capitalization tend to concentrate in different branches of the graph but now, interestingly, BTC is the cryptocurrency displaying the highest centrality. This network is particularly interesting because it shows the direct interactions between pairs of cryptocurrencies that survive after the indirect effects are eliminated. Among these pairs, we can point out the most notable ones. BTC-LTC, where the interaction can be easily understood, considering that LTC is a very popular early fork of BTC. The pair XLM-XRP are two projects related to the Mt. Gox creator, who founded Stellar (XLM) and co-founded Ripple (XRP). These two projects share the same goal of providing blockchain technology advantages to the financial sector. Finally, the chain MAID-DASH-XMR-ETH suggests that the relation MAID-ETH, which can be identified by using correlations, is produced by the indirect interaction with the pair DASH-XMR. This last pair is composed of the two main privacy-oriented cryptocurrencies active in the considered period. All these pairwise direct interactions can be traced back to the network constructed using simple correlations (see Fig 3).

The comparison with the MaST generated using the price returns shows a quite different structure. Some highly ramified clusters are still present, in particular related to BTC and CDN. BTC loses its centrality in favor of DGB but continues connected with coins with small capitalization. Note that effectively DGB is an important coin, one of the earlier second-generation cryptocurrencies. In this case, BTC is also directly interacting with LTC.

**Fig 6:**
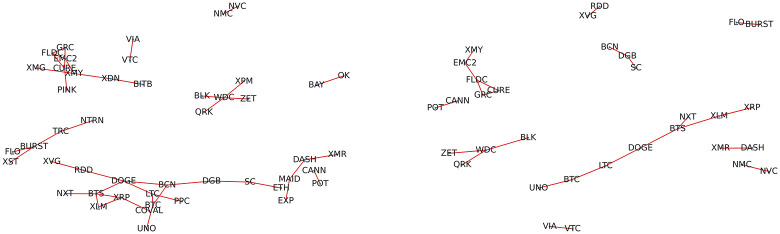
Networks obtained by thresholding the distance generated by the partial correlation matrix for threshold values equal to 1.500 (on the right) and 1.515 (on the left).

Finally, to highlight the simple neighbor structure defined by the most important positive direct interactions between cryptocurrencies and the principal interactions present in the community in Fig 6, we represent the networks obtained by simply thresholding the distance in Eq 5 with different *T* values: nodes *i* and *j* are linked if *d*_*ij*_ > *T*. Note that as we want to highlight interactions with high partial correlations, coins presenting the larger distances are connected. From these networks, we can highlight in a more direct way the most notable relations already captured by the MaST. It is particularly interesting the cluster which contains a branch with the most important (capitalized) first-generation cryptocurrencies (BTC, LTC and DOGE) connected by BTS (the first blockchain to allow for token emission) to the pair XRP-XLM. Another interesting relation is the duplet XMR-DASH.

## Discussion

The results of our analysis of the interaction networks generated using the log-growth rate of the capitalization of the cryptocurrency market are rich with new and interesting results. The correlation matrix of the growth rate displays a very interesting diagonal block structure generated by the capitalization of cryptocurrencies. This fact implies that the network representation of the market presents a clear community structure, with two practically disjointed communities: a smaller one, characterized by currencies of higher capitalization, and a larger one, where the currencies with lower capitalization interact. The correlation values are more pronounced among the most capitalized coins than among the less capitalized ones. The detected interactions are only of the cooperative type, and competitive relations are not relevant. The analyzed set of coins considers the most capitalized and long-lived cryptocurrencies. This fact suggests that longest-lived coins are subjected to cooperative relations and competition relations might be found among the more extinct-prone coins, characterized by short lifetimes. Looking at the hierarchical structure of the community of the more capitalized coins, we can distinguish two clusters: the first is made up of some of the more popular first-generation cryptocurrencies, the second is composed of second-generation cryptocurrencies (more details can be found in S1 Appendix). These results contrast with the network constructed using the price returns of the cryptocurrencies. In this network, the BTC presents a very strong centrality and interacts with a cloud of small-cap coins. Neither the topological structure of the network, nor the correlation values depend on the capitalizations or the prices of the coins. In this case, no disjointed communities sharing common features can be evidenced. The fact that the network represented in terms of the capitalization log-growth rates is so different from the one generated by the return is not so unexpected. Even if capitalization presents an important, but not trivial, dependence on coin price, log- growth rate distributions are in general discernible from the return ones, as can be seen, for example, by looking at the first four moments of the distributions. Moreover, specifically for the most prominent cryptocurrencies, we find low correlations between the time series of the log-growth rate and the return of a given currency. Finally, digital currency issue is very different between cryptocurrencies, affecting the relationship between the two quantities in distinct ways. The critical fact that BTC loses its central position in the log-growth rate-based network, as compared to the conventional one, can be accounted for by the fact that BTC presents an evident prominence among all the cryptocurrencies in terms of returns (for the considered data, the mean daily BTC return is 0.22%, against 0.17% of all other cryptocurrencies). In contrast, in terms of capitalization, BTC accounted for 88% of the total market in 2015, but in 2019, it reduced to 65%. Bitcoin saw a 20-fold capitalization increase, while other currencies saw an 80-fold increase over this period.

The network generated from the partial correlations of the log-growth rate displays a BTC with important centrality. We can highlight interesting correspondences between the detected direct pair interactions and specific features of the related currencies (XLM-XRP, BTC-LTC, DASH-XMR). Moreover, these pairs are coherent with the structures displayed in the correlation network. Direct interactions disclose a core cluster containing a branch with the most capitalized first-generation cryptocurrencies (BTC, LTC and, DOGE) connected by BTS to the pair XRP-XLM. These structures are emergent properties that arise from the multiple interactions among coins and between cryptocurrencies and the financial system, which provides the wealth invested in the market.

The introduction of the correlation analysis based on the log-growth rate, instead of the price returns, generates a network representation of the dataset more structured and detailed in the interconnections between the cryptocurrencies as well as in the overall network topology, which presents a modular structure with compact communities and a rich hierarchy that can be ascribed to different functional groups. In sum, this approach seems to enhance the discriminative potential of the interaction network.

The use of partial correlation analysis allows to highlight the core structure of the pairwise interactions and, compared with the results of the correlation network, shows the robustness and coherence of these approaches. Furthermore, this network of direct interactions should be the starting point for building a possible detailed model with explicit coin interactions for describing the market dynamics.

These results are interesting for the specific case of the cryptocurrency market. The risk strongly depends on the interaction structure of the cryptocurrency system, and its comprehension can be useful for assisting in hedging risks. Starting from the idea that an asset capitalization can serve as a proxy for risk [24, 25], which implies that the smaller the asset, the more it is risky, the identification of the network of directed interactions by using a similarity measure based on the capitalization values, instead of the prices, can give new and important information about the overall risk of very large cryptocurrency market shocks. The analyzed network presents a well-defined clustered structure, with a subset of highly capitalized cryptocurrencies tightly connected and a subset of small capitalized cryptocurrencies loosely connected. This topology suggests less probable widespread contagions, as default cascades should be limited by the disjointed nature of the different network sectors. Moreover, as the low-capitalized (riskier) coins are not strongly interacting coins, it is more difficult that they can drive the market to different, more fragile, states.

These findings are of general importance. Currently, the cryptocurrency market represents less than 1% of the global financial system. However, its size measured by capitalization is similar to the subprime mortgage market that triggered the 2008 crisis. Systemic risk is the risk that one event will cause a succession of other events to cascade, leading to the decline of an industry or the entire economy. So far, we can say that the possibility of systemic risk provided by issues arising from the cryptocurrency market is low. However, as this market is rapidly evolving, cryptoassets can pose problems for financial stability [28]. While the interconnection between unbacked cryptoassets (BTC and ETH, for example) and the traditional financial market has grown substantially, other channels of contagion (use of derivatives and loans to invest in these assets, or lending fiat money using cryptoassets as collateral, for example) have remained low, although these channels of contagion are growing rapidly. Therefore, studies that verify the interconnection between cryptoassets with each other, as well as with the traditional financial system are crucial to subsidize local monetary and financial authorities and avoid a financial debacle.

Finally, our study introduces some new ideas for the study of pairwise interactions which can be useful for biological systems as well. The study of correlations in log-growth rates can bring important advantages over the common method that looks directly at abundances. Moreover, the analysis of the partial correlations results in an important approach that generalizes the standard matrix correlation analysis. The interaction networks produced by this method can be useful for describing modular structure and hierarchy in the communities, which can be related to different species functionalities. On the one hand, this approach is theoretically supported by the Maximum Entropy Framework [5]. On the other hand, by pointing out their equivalence, we show how a standard statistical approach corroborates the Maximum Entropy Framework. This framework, when applied to species abundance, can be related to specific models for the description of the considered population dynamics. The use of the log-growth rate for the detection of interactions can suggest the introduction of new population dynamics models based on these ideas.

## Supporting information

S1 Appendix (PDF)
