# Supplementary material for: Inferring interactions in multispecies communities: The cryptocurrency market case
Source: PLoS One. 2023 Sep 15;18(9):e0291130. doi: 10.1371/journal.pone.0291130 (PMC10503730; doi:10.1371/journal.pone.0291130)
Supplement: S1 Appendix — (PDF) [file pone.0291130.s001.pdf]

Appendix A

The community of the more capitalized cryptocurrencies, as evidenced by the MiST at the top of Fig. 3, contains two interesting clusters: the first with the root corresponding to the BTC, and the second with the root corresponding to the BTS. The following table collects some information about the coins that belong to the first cluster (the BTC cluster):

**Coin Name and Symbol**	**Release**	**Consensus mechanism**	**Purpose**	**Development and Governance**	**Origin**
Bitcoin (BTC)	Jan 2009	Proof of Work (PoW)	peer-to-peer electronic cash	Bitcoin Foundation (modeled on the Linux Foundation) Open Source	
MonaCoin (MONA)	Dec 2013	PoW (ASIC resistant)	peer-to-peer electronic cash (Japanese version of Litecoin)	Community-driven	Litecoin fork (Bitcoin fork), ASIC resistant.
Monero (XMR)	Apr 2014	PoW (ASIC resistant)	Privacy/Anonymity oriented peer-to-peer electronic cash	Monero Research Lab Open Source	Fork from BitMonero
Dash (DASH)	Jan 2014	PoW, Proof of Stake (PoS) for special/faster transactions	peer-to-peer electronic cash with optional privacy (coin mixer)	Block Chain Research Lab (Arizona State University), Decentralized Autonomous Organization (DAO), Open source	Bitcoin fork
Nexus (NXS)	Sep 2014	Tree consensus channels: PoW CPU, PoW GPU and PoS	peer-to-peer electronic cash (Smart contract functionalities added on 2019)	Nexus (DAO implementation is in progress)	Improved version of Bitcoin (quantum-resistant blockchain with tree consensus channels)
WhiteCoin (XWC)	Apr 2014	PoW/PoS hybrid coin now in the PoS phase.	Privacy/Anonymity oriented peer-to-peer electronic cash	Community-driven	Derived from BlackCoin, a fork of Novacoin crypto currency.
Theter (USDT)	Jul 2014	a token (it does not have its own blockchain).	Stable coin	Tether Limited Inc.	Launched on top of the Bitcoin blockchain through the use of the Omni platform. Later it was updated to work on the Ethereum, EOS, Tron, Algorand, and OMG blockchains.
Groestlcoin (GRS)	Mar 2014	PoW and PoS	peer-to-peer electronic cash	Community-driven	Bitcoin fork with an ASIC resistant hashing algorithm (Groestl algorithm).
Litecoin (LTC)	Oct 2011	PoW	peer-to-peer electronic cash	Community-driven	Bitcoin fork , ASIC and GPU resistant.
Syscoin (SYS)	2014	PoW	peer-to-peer electronic cash (in 2020 added smart contract capabilities)	Blockchain Foundry	Litecoin fork
Unobtanium (UNO)	Oct 2013	PoW	peer-to-peer electronic cash (Store of value)	Community-driven	Bitcoin fork
Peercoin (PPC)	Ago 2012	PoS	peer-to-peer electronic cash	Community-driven, stakeholders voting mechanism. Development is coordinated by Peercoin Foundation.	Bitcoin fork<br>First to introduce PoS
BitBay (BAY)	2014	PoS	peer-to-peer electronic cash for a decentralized marketplace	BitBay community	

This second table collects information about the coins that belong to the second cluster (the BTS cluster):

**Coin Name and Symbol**	**Release**	**Consensus mechanism**	**Purpose**	**Development and Governance**	**Origin**
BitShares (BTS)	Jul 2014	delegated proof-of-stake consensus (DPoS)	Global payment network with an integrated decentralized cryptocurrency exchange platform	DAO	Original
MaidSafeCoin (MAID)	Apr 2014	Proof-of-Resource rather than Proof-of-Work (the SAFE Network extends peer-to-peer and distributed hash table technology; consensus is formed from decisions among small self-governing groups of devices or nodes).	Provide a decentralized Internet called 'SAFE network' by using idle computing power to perform routine tasks of Internet servers (e.g. httpd, ssh, scp, ftp, smtp, pop3, imap, etc.).	MAIDSAFE Ltd	Original
Ether (ETH)	Jul 2015	PoW (transitioned to PoS on Sep 2022)	Decentralized virtual machine	Ethereum Foundation, Hyperledger, Nethermind, OpenEthereum, EthereumJS, ConsenSys, Prysmatic Labs, Sigma Prime, Status, ChainSafe, Ledgerwatch, Torquem.	Original
Stellar (XLM)	Jul 2014	Stellar Consensus Protocol (Federated Byzantine agreement, FBA)	Open source, decentralized protocol for digital currency to fiat money low-cost transfers which allows cross-border transactions between any pair of currencies.	Stellar Development Foundation	Original (Share a cofounder with XRP)
Xrp (XRP)	2012	Ripple Protocol Consensus Algorithm (a trust based solution for the Byzantine Generals Problem)	Real-time gross settlement system, currency exchange and remittance network	Ripple (Private company)	Original
Nem (XEM)	Mar 2015	Proof-of-Importance	Decentralized digital platform for decentralized applications	NEM Foundation and open-source NEM project	NEM started in its pre-production stage as a conceptual hard fork of NXT. However, the community behind it decided instead to start a new platform from scratch using totally original code.
Nxt (NXT)	Nov 2013	PoS	Flexible platform around which to build applications and financial services. It has an integrated Asset Exchange (comparable to shares), messaging system and marketplace. Users can also create new currencies within the system. The last major release enabled Multisignature capabilities and a plugin-system for the client.	Jelurida. Secure voting/polling systems	Original
